# Prokaryotic coding regions have little if any specific depletion of Shine-Dalgarno motifs

**DOI:** 10.1371/journal.pone.0202768

**Published:** 2018-08-23

**Authors:** Alisa Yurovsky, Mohammad Ruhul Amin, Justin Gardin, Yuping Chen, Steve Skiena, Bruce Futcher

**Affiliations:** 1 Dept. of Computer Science, Stony Brook University, Stony Brook, New York City, New York, United States of America; 2 Dept. of Molecular Genetics and Microbiology, Stony Brook University, Stony Brook, New York City, New York, United States of America; John Curtin School of Medical Research, AUSTRALIA

## Abstract

The Shine-Dalgarno motif occurs in front of prokaryotic start codons, and is complementary to the 3’ end of the 16S ribosomal RNA. Hybridization between the Shine-Dalgarno sequence and the anti-Shine-Dalgarno region of the16S rRNA (CCUCCU) directs the ribosome to the start AUG of the mRNA for translation. Shine-Dalgarno-like motifs (AGGAGG in *E*. *coli*) are depleted from open reading frames of most prokaryotes. This may be because hybridization of the 16S rRNA at Shine-Dalgarnos inside genes would slow translation or induce internal initiation. However, we analyzed 128 species from diverse phyla where the 16S rRNA gene(s) lack the anti-Shine-Dalgarno sequence, and so the 16S rRNA is incapable of interacting with Shine-Dalgarno-like sequences. Despite this lack of an anti-Shine-Dalgarno, half of these species still displayed depletion of Shine-Dalgarno-like sequences when analyzed by previous methods. Depletion of the same G-rich sequences was seen by these methods even in eukaryotes, which do not use the Shine-Dalgarno mechanism. We suggest previous methods are partly detecting a non-specific depletion of G-rich sequences. Alternative informatics approaches show that most prokaryotes have only slight, if any, specific depletion of Shine-Dalgarno-like sequences from open reading frames. Together with recent evidence that ribosomes do not pause at ORF-internal Shine-Dalgarno motifs, these results suggest the presence of ORF-internal Shine-Dalgarno-like motifs may be inconsequential, perhaps because internal regions of prokaryotic mRNAs may be structurally “shielded” from translation initiation.

## Introduction

Protein synthesis initiates at a “start” codon, typically AUG. However, in random RNA sequence, 1 in 64 triplets is an AUG (considering all three frames). Thus an open reading frame of 1281 nucleotides would contain about 20 AUG triplets, only one of which is the start codon. Furthermore, in prokaryotes, mRNAs are often polycistronic, so that ribosomes have the problem of finding internal start codons as well as the start codon nearest the 5’ end [1]. One mechanism that aids prokaryotic ribosomes in finding the correct start codon is nucleotide complementarity between the tail of the 16S ribosomal RNA (rRNA) and a region immediately upstream of the start codon. Four to ten bases (optimally, 7 bases, [2]) 5’ of a start codon, there is often a four to six nucleotide “Shine-Dalgarno” sequence [1, 3–5], recognized by a complementary sequence near the 3’ end of the 16S rRNA. The single most common Shine-Dalgarno (SD) sequence is AGGAG (Amin et al. submitted), and the 3’ end of many 16S rRNAs contains the sequence CAC**CUCCU**UA-3’OH, which can hybridize with the SD sequence (bold). Hybridization between this anti-Shine-Dalgarno (antiSD) in the tail of the 16S rRNA and the Shine-Dalgarno (SD) motif(s) of the mRNA assists in positioning the ribosome at the correct start codon for initiation of translation [3–6].

Li et al. [7] used ribosome profiling to monitor translation in *E*. *coli*, and saw an enrichment of ribosome footprints that contained SD-like sequences. This originally suggested that the ribosome pauses at SD-like motifs even inside open reading frames. Such internal pauses could be detrimental. Thus, one might expect SD-like sequences to be depleted from open reading frames, and, in *E*. *coli*, this seems to be the case [7].

To follow up on this idea, Diwan and Agashe and Yang et al. [8, 9] did informatic studies on a range of prokaryotes, and, consistent with Li et al, found a slight but statistically significant depletion of SD-like sequences from inside open reading frames. However, the reason for this depletion was unclear. Diwan and Agashe also found a slight but significant depletion of other G-rich sequences that were not SD-like. In addition, they studied a small sample of seven organism that either do not have an antiSD in the 16S rRNA tail, or that do not use the Shine-Dalgarno mechanism. Three of these seven also showed depletion of SD-like sequences. They concluded that “… the frequency of internal SD-like hexamers is governed by multiple factors …”, i.e., not necessarily by the avoidance of complementarity to the 16S rRNA tail.

The finding by Li et al. that ribosomes might pause at internal SD-like motifs, and the further findings by Diwan and Agashe and Yang et al. that SD-like motifs are depleted from open reading frames suggest that the antiSD motifs of the 16S rRNA might “accidentally” hybridize with SD-like motifs inside ORFs; that this hybridization might pause translation; that this would be detrimental; and so natural selection might remove such internal SD motifs.

However, in contrast, recent experiments using ribosome profiling have convincingly argued that the excess SD-containing footprints found by Li et al. [7] arise from a technical artefact during the ribosome profiling procedure, and do not indicate ribosome pausing [10]. Furthermore, experiments intended to detect translational pausing in *E*. *coli* fail to specifically identify SD-like sequences [10–12]. Thus, these experiments suggest detrimental pausing at SD-like sequences inside open reading frames might not occur. But if this is so, what is the explanation of the apparent depletion of SD-like motifs from open reading frames?

Recently, we identified 128 prokaryotes where the 16S rRNA does not contain an antiSD sequence, and where, therefore, the SD mechanism cannot be used for initiation of translation (Amin et al., submitted). Such 16S rRNAs cannot hybridize with internal SD-like motifs. Thus, these organisms should not show any specific depletion of SD-like sequences from open reading frames, and thus should serve as a negative control. However, as we now show here, when the previous analytical methods are applied, these organisms lacking an anti-SD still showed depletion of SD-like sequences from open reading frames, albeit to a somewhat lesser degree. We found the apparent depletion of SD-like sequences found by such methods extends even to eukaryotes, which do not use the SD mechanism, arguing that this depletion is not due to an interaction between the SD and antiSD, and may not be related to translation. Here, we use informatics to further characterize the depletion of SD-like sequences from inside open reading frames of prokaryotes, and ask whether such sequences are specifically depleted. We find that the answer depends critically on exactly how a “SD-like” sequence is defined: sequences rich in G residues are depleted, but this may be independent of whether they can interact with the 3’ tail of the 16S rRNA. We feel the depletion of SD-like sequences is largely (but not entirely) due to a non-specific depletion of G-rich sequences. Together with the results of Mohammad et al. [10] which fail to find translational pausing at internal SD-like sequences, our results suggest there is no significant deleterious interaction between internal SD-like motifs and the 16S rRNA. These results are consistent with the idea that the internal regions of mRNAs may be shielded from translation initiation.

## Results

### Control species with no anti-Shine-Dalgarno in 16S rRNA show depletion of Shine-Dalgarno-like sequences

Diwan and Agashe [8] and Yang et al. [9] recently found that SD-like motifs are depleted from open reading frames of prokaryotyes, perhaps to avoid “accidental” internal binding by the 16S rRNA. However, we recently re-annotated 16S rRNAs from 12,495 prokaryotes (unique taxids), and in doing so found 128 strains or species that lack an antiSD in their 16S rRNAs, and also lack SD motifs in front of their open reading frames (Amin et al., submitted). We realized that such species could serve as a control for the depletion of internal SD-like sequences. That is, if internal SD sequences are depleted because they cause translation problems when they bind to the anti-SD sequence on the 16S rRNA, then species lacking the anti-SD sequence should not suffer such depletion. Diwan and Agashe (2016) had previously used this idea, but had only 7 species to work with.

To test this idea, we applied the methods of Diwan and Agashe using code obtained directly from the authors [8]. The underlying idea of Diwan and Agashe is that if SD-like sequences inside open reading frames are selected against, then this will result in the replacement of certain pairwise combinations of codons by a different but synonymous pair. For example, in *E*. *coli*, the SD is AGGAGG, and this is the sequence produced by an adjacent pair of AGG Arg codons, depending on the frame. Thus, if internal SD motifs are disadvantageous, selection might favor the replacement of AGG AGG by AGG CGC, or CGC AGG, or CGC CGU, etc., all encoding Arg-Arg. The same argument applies to all other possible SD motifs in all three frames. The method of Diwan and Agashe is to consider all the codons in all open reading frames, and randomly shuffle synonomous codons, thereby preserving amino acid sequences, but changing the arrangement of synonomous codons. After such a random shuffle, one can tabulate the frequency of SD-like sequences, and compare it to the real observed frequency before the shuffle. If the *in vivo* observed frequency is lower than the frequency after random shuffles (the “expected” frequency, based on random arrangement, given the amino acid sequences), this implies selection for depletion.

**Fig 1:**
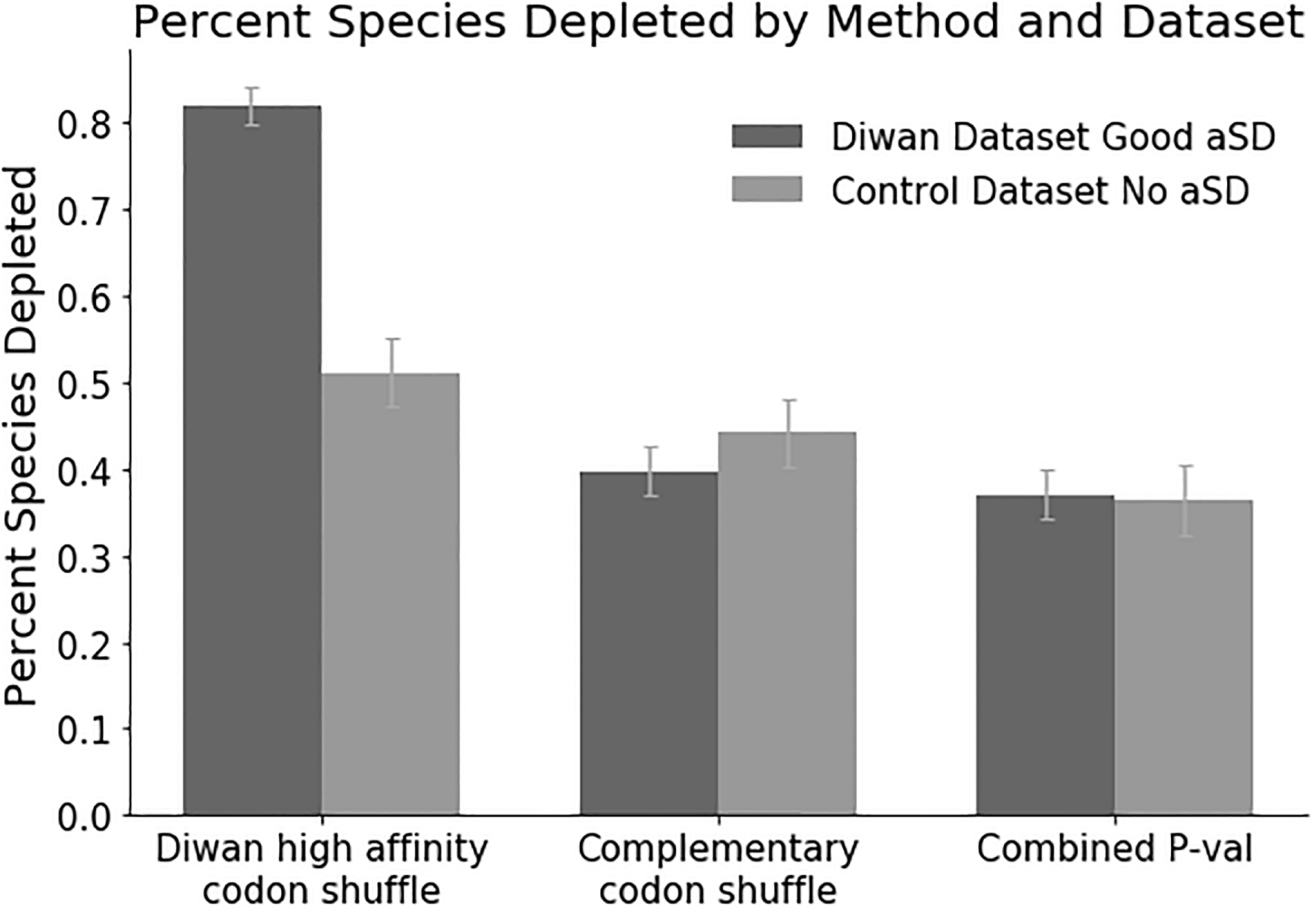
Percentage of species with depleted SD-like sequences. The “Diwan Dataset” comprises the 284 prokaryotes studied by Diwan et al., but filtered for the subset (222) that demonstrably contains a perfect antiSD (CCUCCU) in the tail of the 16S rRNA (Amin et al., submitted). The “Control Dataset” comprises the 128 prokaryotes that lack a CCUCCU antiSD (Amin et al., submitted). The “Diwan high affinity codon shuffle” is the method used by Diwan et al. 2016 [8], and which uses high-affinity hexamers to define SD-like motifs. The “Complementary codon shuffle” is the method used here, using hexamers that are a 5-of-6 match to AGGAGG. The “Combined P-val” is the combination of the complementary codon shuffle with the codon usage metric. Depletion is scored at a p-value of 0.05. Results were not FDR corrected, because the distribution of p-values was not uniform.

We reproduced the results of Diwan and Agashe on the 284 prokaryotes analyzed by them [8]. However, when we applied these methods to the 128 “control” species that lacked an anti-SD, we likewise found that many of these species showed an apparent depletion of SD-like sequences (Fig 1). (Diwan and Agashe obtained a similar result for 3 of 7 similar organisms.) We found significant depletion in 241 of 294 “experimental” species (82%) (284 from Diwan and Agashe, Materials and methods), and significant depletion in 65 of 128 (51%) of the “control” species (Fig 1).

We considered possible explanations for this result. One possibility is that some or all of the 128 “control” species have lost their anti-SD motifs recently in evolution, but that this loss is not yet reflected in open reading frames. However, many of the 128 species come from genera where many (or perhaps all) of the species lack the anti-SD motif, suggesting that the loss of this motif (if it ever existed) was ancient. For instance, we surveyed 66 taxids of the genus Chryeobacterium (Phylum Bacteriodetes, Class Flavobacteriia, Order Flavobacteriales, Family Flavobacteriaceae). Of these 66, for 25 we failed to find an annotated 16S rRNA, and for all of the remaining 41, the anti-SD motif was absent. Similarly, a significant proportion of the species from genus *Mycoplasma* (Tenericutes/Mollicutes/Mycoplasmatales/Mycoplasmataceae), genus *Elizabethkingia* (Bacteriodetes/Flavobacteriia/Flavobacteriales/Flavobacteriaceae) and genus *Candidatus Corsonella* (Proteobacteria/Gammmaproteobacteria/Oceanospirillales/Halomonadaceae) lacked the antiSD.

Furthermore, we reasoned that if a species had lost the anti-SD because of a recent mutation, then SD sequences would still be present in front of many open reading frames. We used the algorithm of Tompa [13] (Methods and materials) to search for conserved motifs in front of open reading frames. For species where the 16S rRNA does contain a CCUCCU antiSD, the Tompa algorithm usually found a SD sequence in front of genes (Amin et al., submitted). However, in contrast, none of the 128 control species lacking a CCUCCU antiSD had a SD in front of their genes (Amin et al., submitted) according to the Tompa method. Again, this strongly argues that the loss of the anti-SD (if it ever existed) was ancient.

#### Eukaryotes show depletion of SD-like sequences

Because the method of Diwan and Agashe found depletion of SD sequences even from “control” species that lacked the antiSD, we did a more extreme test of the same kind by using the Diwan and Agashe method to test for depletion of SD sequences from eukaryotes, which do not use the SD mechanism at all. We tested fungi, which, like bacteria, have a lifestyle strategy of very rapid growth and reproduction. For five of ten fungi we tested, the Diwan and Agashe method showed statistically-significant depletion of SD sequences (Table 1). Furthermore, the average degree of depletion (for eukaryotic species where depletion was seen) was 16%. This was only slightly lower than in prokaryotes, where the average depletion was 21%. These two depletions were not significantly different (p = 0.39, two-tailed t-test). This shows that depletion of these SD-like sequences may be due to forces other than interaction with the 16S rRNA.

**Table 1:** Depletion of SD-like sequences from eukaryotes.

Species	P-value	% Depletion
*S*. *cerevisiae*	0.123	9
*S*. *pombe*	0.175	9
*C*. *albicans*	0.001	30
*P*. *brazilianum*	0.019	9
*S*. *commune*	0.700	0.3
*N*. *crassa*	0.465	-3
*A*. *nidulans*	0.032	6
*C*. *neoformans*	0.206	8
*U*. *maydis*	0.001	23
*A*. *gossypii*	0.002	9

p-values for depletion of SD-like sequences from eukaryotes were calculated as in Diwan and Agashe 2016.

We also looked for SD-depletion in several larger, slower-growing eukaryotes, but, as expected, did not see depletion in those cases.

#### Non-specific depletion of G-rich sequences

We considered several possible reasons this method might be finding depletion of SD sequences even in eukaryotes, and in prokaryotes that do not have an antiSD. Diwan and Agashe define “SD-like” hexamers on the basis of deltaG affinity, not on the basis of complementarity (Table 2). For instance, the hexamer GGGGGG is considered by Diwan and Agashe as a SD-like hexamer (Table 2), even though it has at best a 4 of 6 match to the classic SD sequence (AGGAGG), and even though it is never found by the method of Tompa as an actual SD sequence (Amin et al., in press). Because G:C basepairs have high affinities, the hexamers used by Diwan and Agashe are very G rich. We tried an alternative approach where we considered all hexamers with at least a 5 of 6 match to be SD-like sequences (“complementary” hexamers) (Table 2); these are less G-rich. Although these have a slightly lower average affinity than the hexamers used by Diwan and Agashe, nevertheless biological systems generally show a high degree of specificity. Base complementarity, rather than just affinity, is important in many nucleic acid interactions, such as replication, repair, recombination, transcription, and translation.

**Table 2:** Hexamers used.

Yurovsky	D + A
AAGAGG	
ACGAGG	
AGAAGG	
AGCAGG	
AGGAAG	
AGGACG	
AGGAGA	
AGGAGC	
AGGAGG	AGGAGG
AGGAGT	
AGGATG	
AGGCGG	
AGGGGG	AGGGGG
AGGTGG	
AGTAGG	
ATGAGG	
CGGAGG	CGGAGG
GGGAGG	GGGAGG
TGGAGG	TGGAGG
	GGGGGG
	GGAGGA
	GGAGGC
	GGAGGG
	GGAGGT
	GGGGGT

When we repeated the analysis of Diwan and Agashe using “complementary” hexamers instead of “high-affinity” hexamers (Table 2), results were different (Fig 1). Of the 294 “experimental” species, the percentage showing depletion of SD sequences dropped to about 40%, while of the 128 “control” species the percentage showing SD depletion dropped to about 45% (Fig 1). That is, with this definition of SD-like sequences, there was no difference between the experimental and control species. These results suggest that much of the depletion of G-rich, SD-like sequences seen previously was due to a bias against G-rich sequences of any kind. Indeed, Fig S7 of Diwan and Agashe shows a bias against G-rich sequences that are not SD-like [8]. Given that there is a general bias against G-rich sequences, showing that the depletion of a subset of these G-rich sequences is because they are SD-like becomes problematic. Both our definition and the Diwan and Agashe definition of “SD-like” seem reasonable, and yet they lead to opposite results. Clearly, the definition of a “SD-like” motif is a critical element of the analysis.

#### Codon usage measures

The *E*. *coli* SD is AGGAGG, and, when wholly in the reading frame, this is two consecutive AGG Arg codons. In *E*. *coli*, AGG is a very rare codon, occurring only once per thousand codons, though in some other prokaryotes this codon is much more common (Table 3). The cause-and-effect relationship is unclear: possibly AGG Arg codons are rare because of depletion of SD-like sequences; or possibly SD-like sequences appear to be rare because the AGG codon is rare.

**Table 3:** Codon usage of AGG.

Species	SD	AGG Freq Per 1000
E. coli	AGGAGG	1.0
B. subtiliss	AGGAGG	3.7
T. scotoductus	AGGAGG	15.7
E. rectale	AGGAGG	7.5
R. torques	AGGAGG	3.3
C. aurantiacus	AGGAG	1.4
S. pneumoniae	AGGAG	2.1
M. hungatei	AGGAG	8.4
A. arilaitensis	AGGA	2.2
H. modesticaldum	GGAGG	4.4
M. lacus	GGAGG	10.6
A. aeolicus	GGAGG	26.3
T. neapolitana	GGAGGT	19.5
A. fulgidus	GAGGTG	29.8
T. volcanium	GAGGTG	20.5
P. furiosus	GAGGTG	21.3
P. horikoshii	GAGGTG	31.2
H. mediterranei	GAGGTG	1.1

The frequency of the AGG Arg codon per thousand codons is listed for different species with different functional SD sequences.

Working from this idea, we devised a different and independent measure for depletion of SD-like sequences. If there were selection against SD-like sequences, this should also affect codon frequencies; e.g., AGG should be a less frequent Arg codon than CGC (which, in *E*. *coli*, it is). For organisms with a SD sequence of AGGAGG, there should be selection against the AGG, GGA, GAG, and (again) AGG codons, and they should be less frequent codons. We implemented this as a “Codon Usage Score” as described in Materials and Methods; this score asks whether codons that are subsets of the SD sequence in their species are relatively rare codons.

#### Combining codon shuffling and codon usage finds little or no SD-sequence depletion

Importantly, the “codon usage score” and the “codon pair score” (our name for the codon shuffling method of Diwan and Agashe) are independent of each other, because codon pair scores are normalized for codon usage. To say this in another way, the codon usage score is a measure of how likely it is that a particular codon is used, while the codon pair score is a measure of how likely it is that two given codons are adjacent to one another, given their codon usage. Because of independence, we were able to use the Fisher method to obtain a combined p-value from these two scores. When we use the Fisher combined p-value, the percentage of experimental species showing depletion drops to 37%, and the percentage of control species drops to 36% (Fig 1). That is, again, there is no difference between the experimental and control species, arguing that the depletion seen may not due to any interaction with the anti-SD of the 16S rRNA.

#### Lack of correlation between apparent depletion and strength of SD motif

We used the method of Tompa [13] to find enriched motifs in front of open reading frames in several hundred example prokaryotes (Amin et al. submitted). This is a method for identifying the functional SD sequence in a given species; while most species use AGGAGG or a subset thereof, a substantial minority use a “shifted” motif, such as AAGGA, or GAGGTG (Amin et al., submitted). The Tompa method returns not only a consensus motif, but a Z-score associated with that motif, showing the statistical significance of the motif. We found Z-scores ranging from non-significant (< 5) to as high as 79. A high Z-score occurs when a large fraction of the genes of the organism have a homogeneous motif. Thus, a high Z-score might imply that an organism makes strong use of SD motifs, while a low Z-score might imply that an organism makes little use of such motifs. Conceivably, only organisms strongly dependent on SDs would deplete SD-like motifs from reading frames.

To test this, we correlated the Tompa Z-scores (showing strength of the SD motif) with three kinds of depletion scores: the original Diwan and Agashe codon pair shuffling p-value; our codon pair shuffling p-value using complementary hexamers; and our combined p-value (codon shuffling and codon usage). As shown in Table 4, all three correlations are close to 0 (i.e., no correlation), and only in the third case is the very weak correlation marginally significant. This again suggests a lack of specific depletion.

**Table 4:** Lack of correlation between depletion and strength of SD.

	Correlation	p-value
Diwan high aff. shuffle	0.023	0.695
Complementary shuffle	0.055	0.354
Combined p-value	0.118	0.048

For ~240 species, the p-value of depletion of SD-like sequences was calculated using three different methods, as shown. In addition, for each species, the functional SD sequence was determined by the method of Tompa (1999), and an associated Z-score was obtained. The Spearman correlation was then calculated between the p-values and the Z-scores.

#### Effect size

Beyond the question of whether there is a statistically significant depletion of SD-like sequences (i.e., a small p-value), there is also the question of how big any such depletion might be. Diwan and Agashe (Fig S12) report about 7400 SD-like high-affinity hexamers in species with no depletion, and about 3700 in species with depletion [8]. We estimate that at most, about 3/8 of this (Fig 1, left two bars) is specific depletion. This translates to the loss of very roughly 1400 SD-like hexamers (by the “high affinity” definition), or about one hexamer for every two to three genes in *E*. *coli*, as the maximum size of the effect.

#### Internal SD motifs are not depleted even upstream of start codons

As described above, we found little if any specific depletion of SD-like motifs from inside open reading frames. However, occasionally, an internal SD-like motif would occur just in front of an AUG triplet (in any reading frame). Such an arrangement might, hypothetically, lead not only to ribosome pausing, but to actual (illegitimate?) translation, and this might be highly detrimental. Therefore, such arrangements might be highly depleted. Therefore, we measured the frequency of the SD-like motif AGGAG (the single most common SD sequence) occurring 7 nucleotides (the optimal spacing [2]) in front of AUGs inside open reading frames. We compared this to the overall frequency of internal AGGAG motifs.

**Fig 2:**
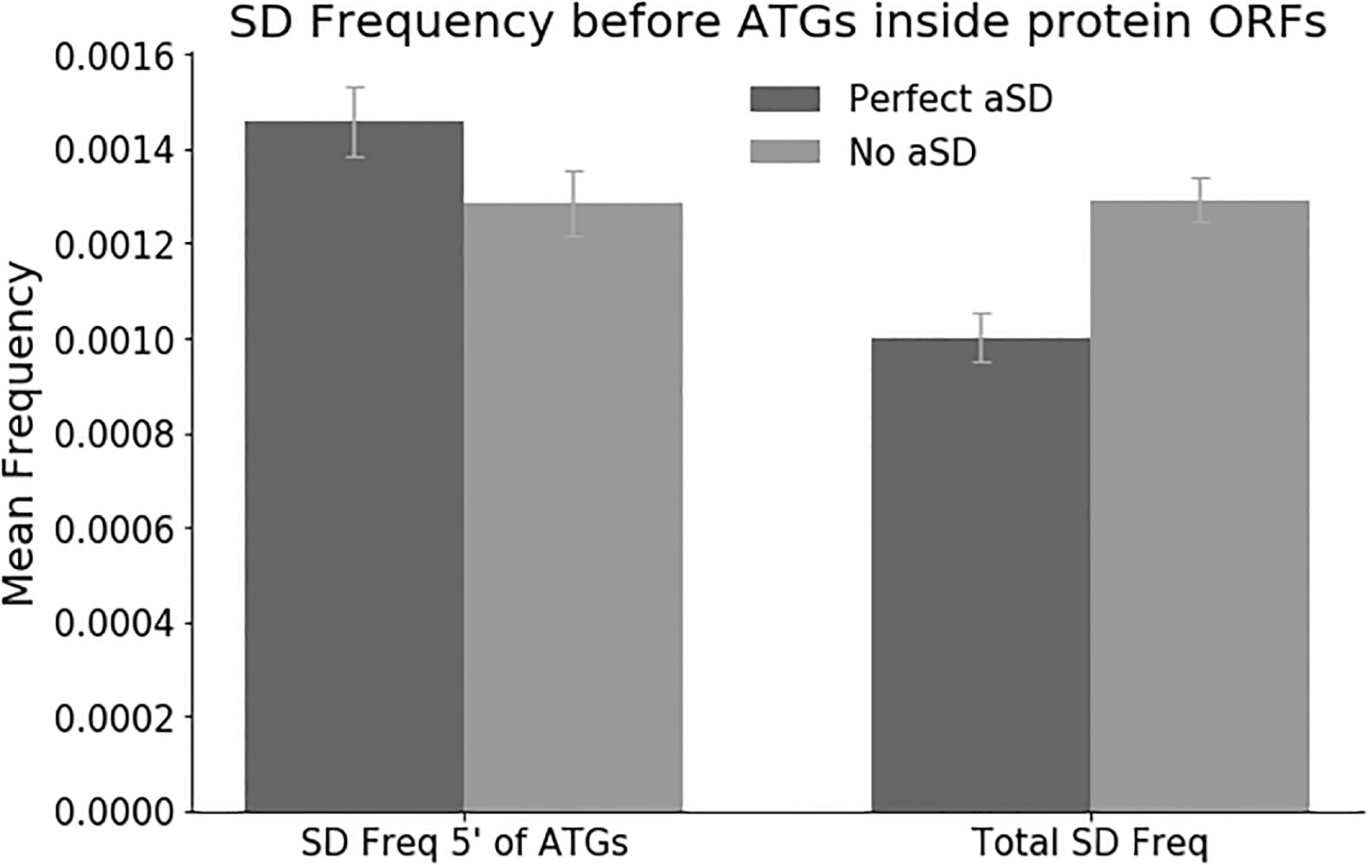
Internal SD motifs are not depleted even when there is a downstream start codon.

Results are shown in Fig 2. As expected, for the control 128 organisms lacking antiSDs, the frequency of internal AGGAG motifs is almost exactly the same regardless of the presence or absence of an AUG seven nucleotides downstream. Surprisingly, however, for organisms with antiSDs in their 16S rRNAs, there was a slight (but statistically significant, p < 10^−11^) increase in the frequency of AGGAG when there was a downstream start codon. We do not understand the significance of this increased frequency, but in preliminary studies it appears that many of the excess AGGAG-AUG combinations are (a) in-frame; and (b) close to the 5’ end of the gene. These facts are consistent with the idea that either the genes in question are mis-annotated, and we are now finding the correct start codons, or that the genes in question are being translated from two different in-frame start codons.

In any case, even in this particularly extreme situation, we fail to see evidence of depletion of internal SD-like motifs, again arguing that there may not be selection against them.

The bars show the frequency of the SD motif AGGAG seven nucleotides upstream of internal AUGs (i.e., **AGGAG**XXXXXXX**AUG**). This was calculated by finding all ORF-internal AUGs (all frames) in all annotated genes in 222 species of bacteria that have 16S rRNAs with antiSDs (10,951,122 ORF-internal AUGs total) or in 128 bacteria that do not have antiSDs in their 16S rRNAs (5,964,321 ORF-internal AUGs total). The frequency of AGGAG seven nucleotides 5’ to these AUGs was then assayed. Separately, we calculated the overall frequency of AGGAG in the same ORFs in these 222 or 128 species. The left pair of bars shows the frequency of AGGAG upstream of AUGs, while the right pair of bars shows the overall frequency of AGGAG. The darker color shows the 222 bacteria with perfect antiSDs in their 16S rRNA, while the lighter color shows the 128 control bacteria with no antiSD. The two frequencies for the “No aSD” bacteria are almost the same and not significantly different. The two frequencies for the “Perfect aSD” bacteria are significantly different (p < 10^−11^). Thus, slight enrichment of SDs is seen in front of AUGs, not depletion.

#### Accelerated evolution of ribosomal protein S1 in organisms lacking an antiSD in their 16S rRNAs

We have found 128 organisms that do not have an antiSD in their 16S rRNAs, and so cannot use the Shine-Dalgarno mechanism to initiate translation. How, then, do these organisms initiate translation? It has been suggested that ribosomal protein S1 is involved in recognition of mRNA [14]. We therefore asked whether there has been accelerated evolution of ribosomal protein S1 in these 128 organisms. For this purpose, we used the dN/dS method (non-synonymous/synonymous substitutions) [15]. As a control, we also looked at dN/dS for ribosomal protein S10, which is not known to be involved in recognizing the mRNA.

**Fig 3:**
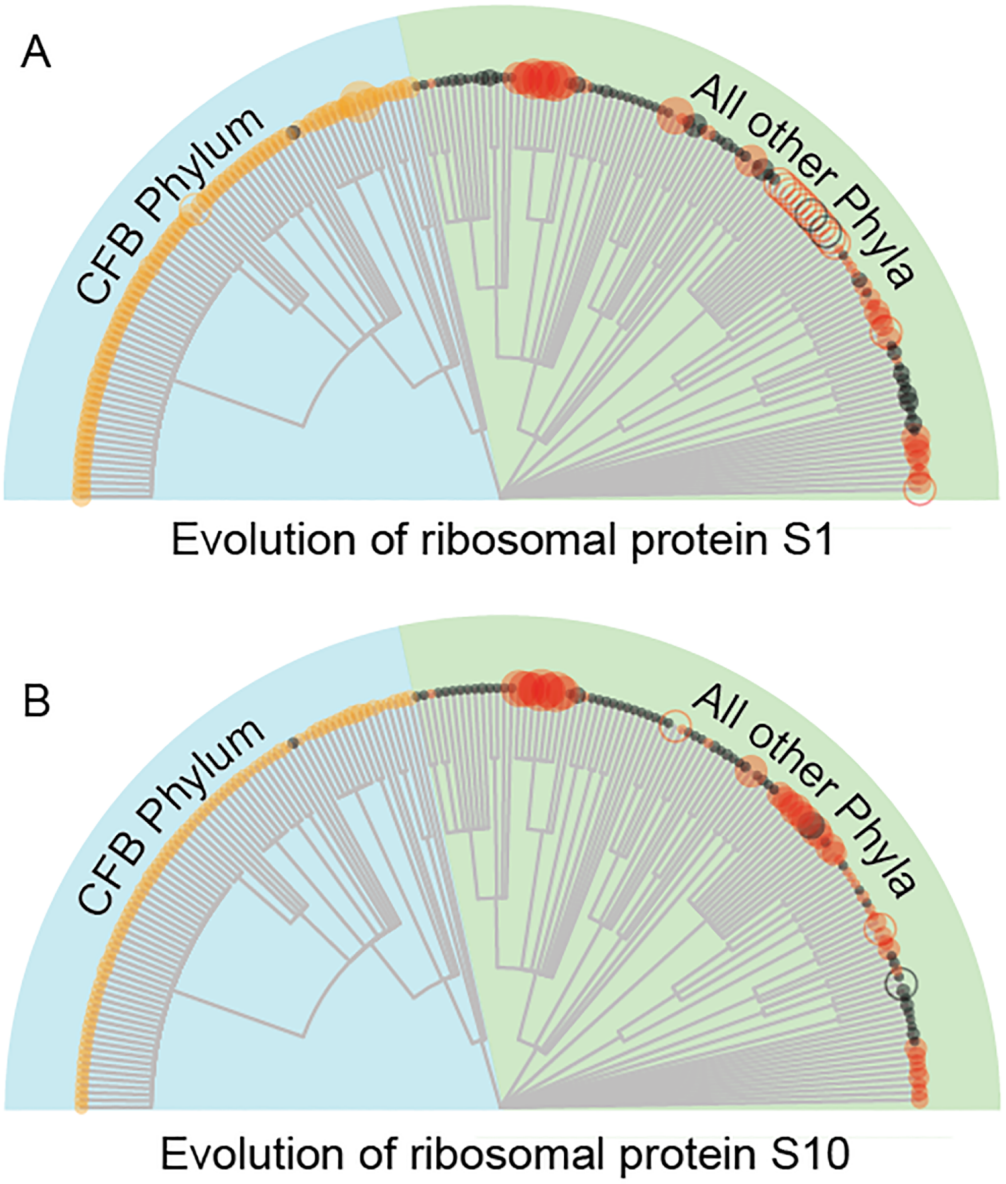
Evolution of ribosomal proteins S1 and S10 in organisms lacking an antiSD. Each circle or dot on the arc of the graph is a query species. The relative dN/dS ratio (versus a control species, which is a close phylogenetic relative that has an antiSD) of the query species is shown by the size of the circle. A large circle indicates a large dN/dS, which indicates rapid directional evolution. Black dots/circles are control species that have an antiSD. Red dots/circles are species lacking an antiSD. Orange dots/circles are species lacking an antiSD, and in the “CFB” (Cytophaga-Flavobacterium-Bacteroides) phylum. In this phylum, species lacking antiSDs are relatively common, and SDs are rare. Empty circles are species where there is no annotated S1 (or, in B, no S10) ribosomal protein; this could mean the protein is present but not annotated, or it could mean the protein is entirely absent.

Results are shown in Fig 3. Species that do have antiSDs (black dots) generally have small dN/dS ratios for both S1 and S10. Species lacking anti SDs, and inside the CFB phylum (Cytophaga-Flavobacterium-Bacteroides) (orange dots/circles) have moderately large dN/dS ratios for S1, and somewhat smaller dN/dS ratios for S10, suggesting somewhat strong directional evolution for S1 after the antiSD is lost. However, since these organisms may not be using a Shine-Dalgarno mechanism to begin with, the interpretation of this is unclear. Outside the CFB phylum, organisms which have lost their antiSD (red circles) have large dN/dS ratios, indicating strong directional evolution. However this evolution is about equally strong for both ribosomal protein S1 and S10, which may argue more for a wholesale change in the ribosome, than for specific changes in S1. Interestingly, quite a few of the red circles are empty, denoting a lack of annotated S1, which could mean that ribosomal protein S1 becomes dispensable after the antiSD is lost.

## Discussion

Diwan and Agashe, and Yang et al. recently analyzed the frequency of SD-like sequences, and concluded that most prokaryotes are depleted for such sequences [8, 9]. We reproduced the results of Diwan and Agashe, finding about 80% of prokaryotes have significant depletion by their methods. However, the reason for this depletion is unclear. Because we have recently identified 128 prokaryotic species that lack an anti-SD in their 16S rRNAs, we were able to use these as a control group. Perhaps surprisingly, we found that previous methods still find depletion of SD-like sequences in about half of these “control” species, and also in about half of a small sample of eukaryotes. The fact that depletion is found so often even in species lacking an anti-SD suggests that some or much of this depletion may not be specific to SD-like sequences.

The SD-like sequences used by Diwan and Agashe are exceptionally G-rich, and there is a non-specific depletion of G-rich sequences in prokaryotes (Diwan and Agashe, Fig. S7) [8]. We used an alternative definition of “SD-like”, namely hexamers that have at least 5 of 6 base complementarity to the consensus SD AGGAGG; these are less G-rich. With this definition, we could find no specific depletion of such hexamers. Because the answer to the question of whether SD sequences are depleted depends so much on the definition of “SD-like”, and because G-rich sequences are non-specifically depleted, the question becomes both problematic and semantic. But in any case, for many prokaryotes, the degree of depletion—the effect size—is rather small, and not obviously different from the depletion of similar sequences in eukaryotes.

Possibly for some prokaryotes, significant depletion occurs in highly-expressed genes only [9]. Additionally, it may occur mainly in faster-growing bacteria [8]. Our analysis does not address either of these possibilities.

The finding of Li et al. [7] that ribosomes pause at SD-like motifs inside genes, combined with the findings from Diwan and Agashe and Yang et al. [8, 9] that ORFs are depleted of SD-like motifs, contribute to a narrative suggesting that internal SDs bind and pause ribosomes somewhat efficiently; that these pauses are detrimental; and so there is selection against internal SD motifs. However, there is now an alternative narrative, that internal SDs do not pause ribosomes [10–12], and to the extent that SD-like motifs may be depleted, this is not because they are Shine-Dalgarnos, and not because of any interaction with the antiSD of the 16S rRNA (this work). This alternative narrative is most consistent with the idea that SD-like motifs inside ORFs do not efficiently interact with antiSDs, and this may mean that internal regions of mRNAs are not very accessible for the initiation of translation. This is supported, for instance, by the fact that internal SDs are not depleted even when they are 7 nucleotides upstream of an AUG triplet (Fig 2).

Continuing with this last idea of the possible inaccessibility of internal regions of mRNAs, we are struck by the fact that a significant number of prokaryotes do not seem to use an SD sequence at all, and even in those that do, it is only for some genes [1, 16–19]. This is despite that fact that these prokaryotes have polycistronic mRNAs, where translation must initiate at several different start codons on the same mRNA. This raises the issue of what other mechanisms are used to find the correct site for initiation translation, and what determines which of these mechanisms predominates. Other proposed mechanisms include recognition of the 5’UTR of prokaryotic open reading frames by ribosomal protein S1 [14, 18, 20, 21] and a mechanism for recognizing leaderless mRNAs [18, 22, 23]. In addition, several workers have stressed the importance of mRNA secondary structure, which may shield internal SDs and non-start AUG codons from the ribosome, while an absence of secondary structure near genuine SDs and start codons may allow an interaction with the ribosome [24–31]. Such internal shielding is highly consistent with many of our results. It may be that one of these mechanisms will prove to be the primary mechanism for determining initiation of translation, and the Shine-Dalgarno mechanism may be only a helpful secondary mechanism.

## Materials and methods

Unless explicitly stated, all calculations were done using custom Perl, Python, R, and Mathematica scripts. Scripts of Diwan and Agashe were obtained from the authors [8] and were used as described.

### Sequence data

All genome sequences were acquired from ftp.ncbi.nih.gov.

We pre-processed open reading frames to discard those which did not begin with the start codon, did not end on a stop codon, or had stop codons in frame in the middle.

### Shine-Dalgarno identification

The putative SD sequences for each of the bacteria were discovered using the algorithm of M. Tompa [13]. We acquired the program source code from the author and followed the attached instructions. We pre-processed the data to collect short DNA sequences upstream of each gene. We then ran RBSidentify, considering patterns of length 7, and allowing for 1 mis-match. This program outputs the z-scores listing the probability of each 7-mer to occur randomly in the given list of upstream sequences. We only consider the bacteria where the top 20 7-mers have a z-score greater than 8, to ensure significance based on the simulated data z-scores. We then ran RBSprofile on the top 20 7-mers to identify the most significant positions in the unified motif, measured by the relative entropy, reading off the putative SD sequences from the generated matrix.

To further verify the putative SD sequence for each bacterial genome, we checked that its complement exists near the 3' end of the ribosome’s 16S rRNA sequence. If the complement did not exist near the end of the original annotated 16S rRNA, we asked whether the annotated 3’ end could be extended along the genome sequence and still maintain high homology to the *E*. *coli* 16S RNA. In many cases it could, suggesting misannotation of the 3’ end in the database. In these cases, we usually found the putative SD in the corrected (extended) sequence (Amin et al., submitted).

### Simple SD depletion measure

If our hypothesis is that *E*. *coli*, for example, should avoid AGGAGG in coding regions, the most direct approach is to simply count the occurrences of AGGAGG in coding regions, and compare that to the number expected from nucleotide frequencies in coding regions. For each organism, we count A, T, C, and G in coding regions and calculate the number of SD sequences expected in any frame. We divide observed SD motifs by the expected number and take the natural logarithm, to make this measure comparable to codon pair bias.

### Codon usage score

For each organism, we consider every possible nucleotide string of the same length as that organism’s SD. E.g., for *E*. *coli*, with a 6-mer SD, we have 4096 such strings. For each such candidate string, we find all the codons that are sub-strings (e.g., for *E*. *coli* AGGAGG, there are four substrings, AGG, GGA, GAG, and AGG). For 5-mer SD sequences, there are three substrings; for 4-mer SD sequences, there are two substrings. For each of these codons, we look up the frequency of that codon in filtered coding regions. E.g., for *E*. *coli* AGG, the frequency of usage is 0.001. We include the frequency of usage of each stop codon in the calculation, if a stop codon happens to be part of the motif string. Now for each candidate string, we calculate a measure: the average of the codon frequencies of the 4, 3, or 2 codon substrings.

For each organism, we get a distribution, which has 4096, or 1024, or 256 numbers in it. Now we calculate the above measure for the actual SD sequence of each organism, and see where in the distribution the actual measure falls. This directly gives us a p-value (one-tailed) for the SD motif depletion.

### Codon pair bias (codon shuffling)

We calculated the codon pair scores for each of the bacterial genomes using the method described in Coleman et al. [32]. The score is the natural logarithm of the observed number of codon pairs in the coding regions divided by the number expected based on codon frequencies. For each bacterial genome we selected the codon pairs that are 5 out of 6 and a 6 out of 6 match to the respective SD sequence (or 4 out of 5 and 5 out of 5, etc.). To calculate the significance of depletion of these codon pairs, we performed a one-tailed t-test between all codon pair scores and the scores for codon pairs with 5/6 and 6/6 matches to the SD. This procedure is very similar to the codon shuffling procedure of [8]
